# Histopathological Characteristics and Prognosis of Malignant Acral Melanomas in Pakistani Patients

**DOI:** 10.7759/cureus.33617

**Published:** 2023-01-10

**Authors:** Anum Shahid, Madiha Syed, Sarah Jamshed, Umer Sheikh, Asif Loya, Usman Hassan, Mudassar Hussain, Maryam Hameed, Sajid Mushtaq

**Affiliations:** 1 Histopathology, Shaukat Khanum Memorial Cancer Hospital and Research Centre, Lahore, PAK

**Keywords:** metastasis, recurrence, prognosis, foot, melanoma

## Abstract

Background

Malignant melanoma is a common cancer in Scandanavian countries due to increased exposure to ultraviolet light. Very limited data is available on malignant melanomas in Pakistani population and further studies are needed to determine its incidence in our population.

Objective

The main objective of our study was to determine histopathological characteristics and prognosis of malignant foot melanomas in Pakistani patients.

Material and methods

After approval by the Institutional Review Board, we performed a retrospective study of 59 consecutive cases of malignant acral melanoma from the year 2016-2019. The follow-up of in-house cases was available in hospital archives. The follow-up of diagnostic patients was done through direct communication. The histological features were assessed, and the prognosis was determined in terms of recurrence, metastasis, and death.

Results

The main histological features assessed were Breslow thickness <1 (n=3), >1-2 (n=9), >2-4 (n=12), >4 (n=36), ulceration was present in 65% (n=39), and pathological stage 1 (n=3), stage 2 (n=9), stage 3 (n=12) and stage 4 (n=36). The margin was involved in 28.3% (n=17) cases. Recurrence was observed in 47.4% (n=28), metastasis in 55.9 % (n=33), and death was observed in 49.1% (n=29). The mean follow-up duration of 3.4 years ± 0.20 (Range 3 to 6 years). The recurrence-free survival was 2.9 ± 0.24 years, metastasis-free survival was 2.8 ± 0.237 years, and disease-specific survival was 3.4 ± 0.203 years.

Conclusion

Malignant acral melanoma is fatal with high mortality rates. In our part of the world, acral melanoma has poor prognosis compared to non-acral melanomas. When compared with acral melanomas in other parts of the world prognosis is even worst. Early diagnosis and treatment are crucial in terms of patient management.

## Introduction

The incidence of malignant melanoma by race is 1.9/100,000 in Hispanics, 9.2/100,000 in whites, and 0.7% to 1.2/100,000 in blacks and Asian [1]. In Europe, the incidence rate is about <25 new cases of melanoma per 100,000 population [2]. In the United States, the incidence rate is 30 per 100,000 [2]. The incidence rate is extremely high in Australia, as it reaches 60 cases per 100,000 [3]. According to data by GLOBOCAN 2020 incidence of malignant melanoma in India is 3916 cases/per year. Melanoma is the 32^nd^ most common malignancy in Pakistan. Very limited data is available regarding melanomas in the Pakistani population, and further studies are needed to determine its incidence.

Several risk factors play a significant role in the development of cutaneous melanomas. These can be divided into environmental factors and genetic factors. Skin pigmentation has a significant influence on skin susceptibility to malignant change. Melanocortin 1 receptor (MC1R) is a cell surface receptor in melanocytes, and it induces pigment production [4]. There are many polymorphisms of the MC1R gene. Skin phenotypes such as red hair and fair complexion are primarily determined by the MC1R gene. Fair skin with low pigmentation shows increased sensitivity to ultraviolet (UV) light and an increased risk of associated melanoma. In addition to characterizing the phototype, melanin plays a vital role in defending melanocytes and keratinocytes from UV light [5]. Phototypes I and II are more susceptible to UV damage and are at higher risk of developing melanocytic and keratinocyte cancers. Red hair phenotype acquired melanocytic nevi and melanocyte-stimulating hormone receptor (MC1R) alleles all independently increase melanoma risk [1].

Melanoma in non-sun-exposed areas is more common in Asians than Caucasians. The data regarding melanomas in non-sun-exposed areas in the Asian population, especially the Pakistani population, is very limited. This motivated us to conduct research on acral melanomas in the Pakistani population.

## Materials and methods

We performed a retrospective study of 60 cases of malignant acral melanoma from the year 2016-2019. The follow-up of in-house cases was available in the hospital database. The diagnostic patients were directly communicated with for follow-up. Our study included excision specimens of primary foot melanomas only. Incisional biopsies, melanoma in situ only, prior history of therapy or surgery, and mucosal and non-acral melanomas were excluded from the study. The study was approved by the Institutional Review Board.

The slides were reviewed by a consultant and fellow surgical pathologists. The histopathological parameters like ulceration, Breslow thickness, Clark’s level, tumor-infiltrating lymphocytes (TILs), tumor regression, growth phase, and margin status were assessed. The pathological tumor stage was assigned according to CAP protocol 2022. Survival analysis was done in terms of recurrence and metastasis. Death was considered a clinical endpoint in our study. Recurrence was defined as any reappearance of the disease after treatment.

Histopathological features were analyzed using I BM Corp. Released 2011. IBM SPSS Statistics for Windows, Version 20.0. Armonk, NY: IBM Corp. The Chi-square test was used for categorical variable analysis. The Kaplan-Meier method was used to generate survival curves.

## Results

Our study includes a total of 59 cases of acral melanomas with a mean follow-up duration of 3.4 years ± 0.20 (Range 3 to 6 years). The mean age of patients was 53 ± 1.741 years (Range 24-84 years), with 33 (55%) males and 27 (45%) females. Twenty-eight (46.7%) cases of malignant melanoma involved the right foot, and 21 (35%) occurred in the left foot. The heel was the commonest site 17 (28.3%) (Table 1).

**Table 1:** Demographics

Parameters	Frequency (n)	Percentage (%)
Gender		
Male	33	55
Female	27	45
Site On Foot		
Big toe	1	1.7
Medial side	1	1.7
Sole and toes	1	1.7
Dorsum	3	5
Plantar	3	5
Toes	5	8.3
Soles	11	18.3
Heel	17	28.3
Not specified	18	30
Laterality		
Right	28	46.7
Left	21	35
Not Specified	11	18.3

The histological parameters were assessed according to CAP protocol 2021 (Table 2). 

**Table 2:** Histological parameters

Parameters	Frequency (n)	Percentage (%)
Histological type		
Superficial spreading and nodular	2	3.3
Superficial spreading	4	6.7
Nodular type	25	41.6
Acral lentiginous	15	25
Not specified	14	23.3
Margin status		
Involved	17	28.3
Uninvolved	43	71.7
Satellite/ In-transit metastasis		
Present	3	5
Absent	57	95
Clark’s Level		
I	0	0
II	8	13.3
III	6	10
IV	20	33.3
V	26	43.3
Ulcer		
Present	39	65
Absent	21	35
Breslow’s Thickness		
<1	3	5
>1-2	9	15
>2-4	12	20
>4	36	60
TILS’s		
Brisk	3	5
Non-brisk	11	18.3
Absent	46	76.7
Growth phase		
Vertical	54	90
Radial	6	10
Tumor Regression		
Present	2	3.3
Absent	58	96.7
pT Stage		
1a	2	3.3
1b	1	1.7
2a	5	8.3
2b	4	6.7
3a	6	10
3b	6	10
4a	8	13.3
4b	28	46.7

The commonest histological type was nodular melanoma at 41.6% (n=25) with an involved margin of 28.3% (n=17). Other histological features included ulceration present in 65% (n=39), Breslow thickness <1 (n=3), >1-2 (n=9), >2-4 (n=12), >4 (n=36), Clark’s level I 0% (n=0), level II 13.3% (n=8), level III 10% (n=6), level IV 33.3% (n=20), and level V 43.3% (n=26), satellite/in-transit metastasis was present in 5% (n=3), tumor-infiltrating lymphocytes (TILS) were present in 14 cases brisk 5% (n=3) and non-brisk 18.3% (n=11), vertical growth phase was identified in 90% (n=54) and radial in 10% (n=6) and tumor regression occurred in 3.3% (n=2). The pathological stage included stage I 5% (n=3), stage II 15% (n=9), stage III 20% (n=12), and stage IV 60% (n=36).

We stratified our data according to the pT stage. pT1 included 5% cases (n=3), pT2 15.25% cases (n=9), pT3 20.34% cases (n=12), and pT4 59.3% cases (n=35).

Recurrence was observed in 3.6% cases (1/3) in pT1, 3.6% cases (1/9) in pT2, 25% (7/12) cases in pT3, and 67.9% (19/35) cases in pT4 with p-value 0.04.

Metastasis was observed in 0% cases (0/3) in pT1, 12.1% cases (4/9) in pT2, 15.2% cases (5/12) in pT3, and 72.7% cases (24/35) in pT4. The p-value of 0.05 showed statistically significant results.

Death was observed in 0% cases (0/3) in pT1, 6.9% cases (2/9) in pT2, 10.3% cases (3/12) in pT3, and 82.8% cases (24/35) in pT4. The p-value of 0.01 showed statistically significant results.

**Figure 1:**
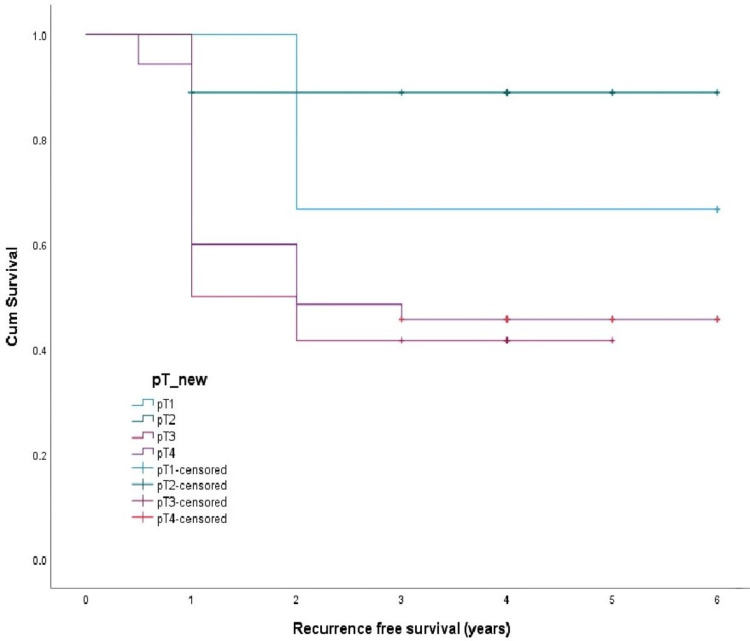
Recurrence-free survival

**Figure 2:**
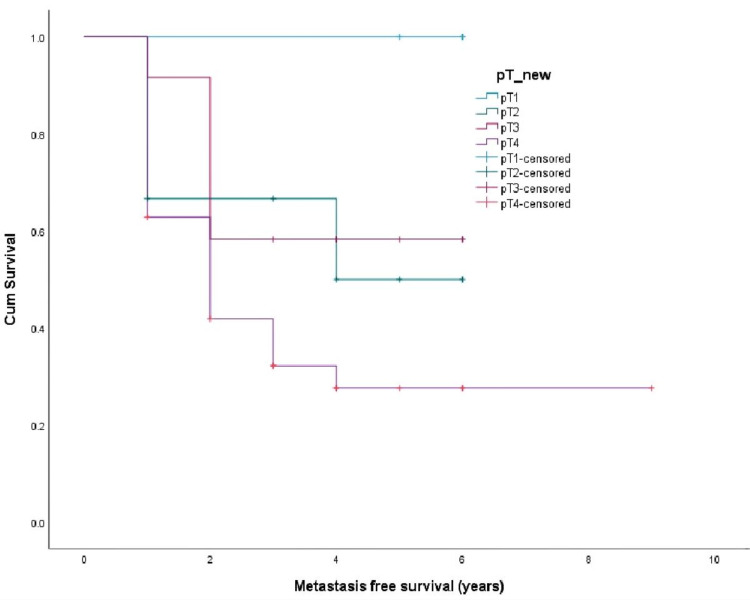
Metastasis-free survival

**Figure 3:**
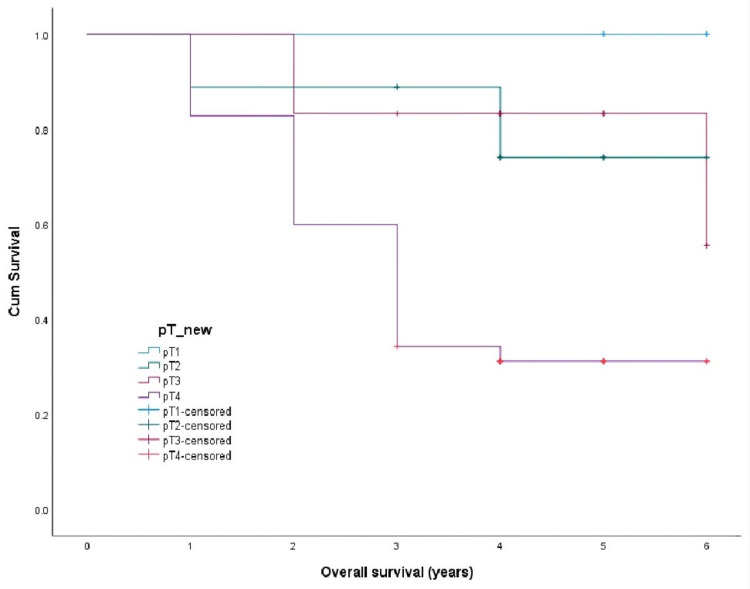
Overall survival

The survival curves were drawn by the Kaplan Meir method and compared by Log Rank Test. The overall recurrence-free survival was 2.9 ± 0.24 years (Figure 1), metastasis-free survival was 2.8 ± 0.237 years (Figure 2), and disease-specific survival was 3.4 ± 0.203 years (Figure 3). Recurrence-free survival (p-value 0.14), metastasis-free survival (p-value 0.05), and overall survival (p-value 0.04) were worst for pT4 tumors compared to pT1-3.

## Discussion

Cutaneous melanoma is the commonest disease in Scandinavian countries. The disease is commonly associated with a fair-skinned population and increased exposure to ultraviolet light. There is limited data available regarding malignant melanoma in South Asian countries, especially Pakistan. This motivated us to conduct research to determine histopathological characteristics and prognosis of malignant acral melanomas in Pakistani patients. The foot is the commonest site of melanomas in the Pakistani population. In contrast to cutaneous melanomas on other anatomical sites, volar sites are of keen interest due to limited sun exposure. The pathogenesis of malignant melanoma on the plantar surface remains unclear. It has been hypothesized by Jung H et al. [6] that trauma is a pre-disposing factor in acral melanomas. This hypothesis has been supported by the study of Ishihara et al. [7]. Mechanical stress is another causative factor highest on the front and heels compared to other areas on the sole. Saida et al. [8] hypothesized in their study that volar skin is hypopigmented even in black skin and is a predisposing factor for malignant melanomas. The reason is that melanin molecules act as blockers of free radicals generated locally by inflammation [9].

We retrospectively surveyed 60 cases of acral melanomas (AM) and compared our study results with another study of 28 non-acral melanoma cases (NAM) of our institution published in 2020 [10]. The histopathological characteristics and oncological outcomes of AM and NAM are shown in Table 3.

**Table 3:** Comparison of foot and non-foot melanomas

Parameters	Foot melanoma Frequency (n)	Foot melanoma Percentage (%)	Non-foot melanoma Frequency (n)	Non-foot melanoma Percentage (%)
Gender				
Male	33	55	16	57.1
Female	27	45	12	42.9
Histological type				
Superficial spreading and nodular	2	3.3	-	-
Superficial spreading	4	6.7	1	3.5
Nodular Type	25	41.6	6	21.4
Acral lentiginous	15	25		
Un-classified	14	23.3	21	75
Margin status				
Involved	17	28.3	5	18.5
Un-involved	43	71.7	23	82.1
Clark’s Level				
I	0	0	1	3.6
II	8	13.3	1	3.6
III	6	10	1	3.6
IV	20	33.3	13	46.4
V	26	43.3	5	17.9
Ulcer				
Present	39	65	15	53.5
Absent	21	35	13	46.4
Breslow Thickness				
<1	3	5	1	3.5
>1-2	9	15	10	35.7
>2-4	12	20	10	35.7
>4	36	60	7	25
pT Stage				
i	3	5	1	3.6
ii	9	15	3	10.7
iii	12	20	4	14.3
iv	36	60	13	46.4
Lost	-	-	7	25
Recurrence				
Present	28	47.4	17	60.7
Absent	31	52.5	11	39.2
Lost	1	1.7		
Metastasis				
Present	33	55.9	14	50
Absent	26	44	14	50
Lost	1	1.7	-	-
Death				
Dead	29	49.1	6	21.4
Alive	30	50.8	18	64.3
Lost	1	1.7	4	14.3

The AM patient group comprised 33 males and 27 females with a mean age of 53 ± 1.741 (Range 24-84 years). The NAM patient group, on the other hand, comprised 16 males and 12 females with a mean age of 46.5 ±15.9 (Range 20-80 years). The mean age of patients with AM was higher than that of NAM. The mean age in our study was 53 ± 1.741 years compared to NAM 46.5 ±15.9 years. There was male predominance in both studies. The heel was the most common site in the foot, 28.3% (n=17), and the lower limb, 46.4% (n=13) in NAM.

The morphological characteristics of AMs and NAMs showed that AMs were more likely to display nodular histological type than NAM (Table 3). Furthermore, AMs were more likely to show ulceration 65% (n=39) than NAMs 53.5% (n=15). The incidence of Breslow’s thickness >4 was significantly higher in AMs than in NAMs (60% vs. 25%). Regarding completeness of excision. The margin was involved in 28.3% (n=17) cases of AMs and 18.5% (n=5) cases of NAMs. The percentage of AJCC stage IV was higher in AMs compared to NAMs, 60%, and 46.4%, respectively. 

The survival analysis showed higher rates of metastasis in AMs, 55.9% (n=33), compared to NAMs, 50% (n=14). Recurrence was observed in NAMs 60.7% (n=17) and AMs 47.4% (n=28). Death due to disease occurred in 49.1% (n=29) AMs and 21.4% (n=6) NAMs.

The overall comparison of histopathological features and survival analysis of AMs and NAMs showed poor prognosis of AMs.

We also compared our results with acral melanomas in other populations. Compared to acral melanomas in Taiwanese [11], Korean [12], and Chinese patients, [13-14] acral melanomas in Pakistani patients were pre-dominantly nodular subtype with higher rates of ulceration (65% vs. 32.7% vs. 42.3% vs. 47.9%) and Breslow thickness >4mm (60% vs. 25.5% vs. 32.9% vs. 47.9%). The overall survival was worst for the Pakistani population, 50.8%, followed by the Chinese population, with an overall survival rate of 65.6% in non-stress bearing and 55.8% in stress-bearing sites of foot [13-14].

Like other studies, our study is not without limitations. The main limitation is the late stage at the time of presentation, which is a major contributing factor to a dismal prognosis. To get insights into the behavior of acral melanomas, it is important to have larger prospective studies, including early-stage lesions, to determine an accurate prognosis.

## Conclusions

Based on our study results, we conclude that the histopathological characteristics and prognosis of AMs is worst than NAMs in Pakistani patients. Also, AMs in Pakistani patients showed the worst prognosis compared to acral melanomas in other parts of the world. One of the major factors associated with poor prognosis is the late stage at the time of presentation. Early diagnosis and prompt management may play a significant role in improving survival.
